# Reduction in the Level of Plasma Mitochondrial DNA in Human Diving, Followed by an Increase in the Event of an Accident

**DOI:** 10.3389/fphys.2018.01695

**Published:** 2018-11-29

**Authors:** Jean-Eric Blatteau, Sandrine Gaillard, Sébastien De Maistre, Simone Richard, Pierre Louges, Emmanuel Gempp, Arnaud Druelles, Henri Lehot, Jean Morin, Olivier Castagna, Jacques H. Abraini, Jean-Jacques Risso, Nicolas Vallée

**Affiliations:** ^1^Institut de Recherche Biomédicale des Armées, Equipe Résidante de Recherche Subaquatique Opérationnelle, Département Environnement Opérationnel, Unité Environnements Extrêmes, Toulon, France; ^2^Hôpital d’Instruction des Armées – Service de Médecine Hyperbare et Expertise Plongée, Toulon, France; ^3^Biotech Services, Université de Toulon, Toulon, France; ^4^Mediterranean Institute of Oceanography, Université de Toulon, Toulon, France

**Keywords:** dive, ischemia, systemic inflammation, stroke, inert gas, PAMPS, TLR, neutrophil

## Abstract

Circulating mitochondrial DNA (mtDNA) is receiving increasing attention as a danger-associated molecular pattern in conditions such as autoimmunity or trauma. In the context of decompression sickness (DCS), the course of which is sometimes erratic, we hypothesize that mtDNA plays a not insignificant role particularly in neurological type accidents. This study is based on the comparison of circulating mtDNA levels in humans presenting with various types of diving accidents, and punctured upon their admission at the hyperbaric facility. One hundred and fourteen volunteers took part in the study. According to the clinical criteria there were 12 Cerebro DCS, 57 Medullary DCS, 15 Vestibular DCS, 8 Ctrl+ (accident-free divers), and 22 Ctrl- (non-divers). This work demonstrates that accident-free divers have less mtDNA than non-divers, which leads to the assumption that hyperbaric exposure degrades the mtDNA. mtDNA levels are on average greater in divers with DCS compared with accident-free divers. On another hand, the amount of double strand DNA (dsDNA) is neither significantly different between controls, nor between the different DCS types. Initially the increase in circulating oligonucleotides was attributed to the destruction of cells by bubble abrasion following necrotic phenomena. If there really is a significant difference between the Medullary DCS and the Ctrl-, this difference is not significant between these same DCS and the Ctrl+. This refutes the idea of massive degassing and suggests the need for new research in order to verify that oxidative stress could be a key element without necessarily being sufficient for the occurrence of a neurological type of accident.

## Introduction

An alteration in the number of copies of circulating mitochondrial DNA (mtDNA) has been observed in many human pathologies (Zhang et al., 2010a,b; Itagaki et al., 2015; Leandro et al., 2015). In particular its detection serves to assess the cell damage induced by oxidative stress, but a significant inflammatory role is starting to be attributed to it (Itagaki et al., 2015 Leandro et al., 2015). Its detection seems particularly interesting in the context of decompression sickness (DCS), particularly of neurological type, occurring after a dive.

The practice of scuba diving requires inhalation through a regulator of pressurized gaseous mixtures inducing the presence of (i) dissolved gases throughout the organism and (ii) of circulating bubbles following desaturation. The presence of circulating bubbles in the blood vessels may be detected even if the diver has observed the decompression tables, these last are supposed to ensure a safe return to the surface (i.e., without generating bubbles). The bubbles remain the primum movens at the origin of the decompression accident which regularly degenerates into DCS. It seems that the inflammatory and ischemic phenomena are the result of the reaction of the bubbles with the body (Brubakk et al., 2003a), but the mechanisms remain uncertain. In its severest forms DCS extends systemically and may induce severe neurological deficits, including paralysis or even death (Vann et al., 2011).

Treatment of decompression accidents is a medical emergency, which has empirically combined the inhalation of normobaric oxygen at the onset of the first signs, followed by oxygen therapy in a hyperbaric enclosure, with the administration of drug treatment with rheological and anti-inflammatory intent. The aim of the various treatment methods is to combat or limit the consequences of the bubble phenomenon (Ersson et al., 1998; Nossum et al., 1999, 2002), i.e., tissue hypoxia caused by the interruption to the arterial or venous circulation following a gas embolism, as well as cytotoxic phenomena of DCS. However, after treatment of the civilian or military divers, 20–30% of patients present permanent sequelae, half of which cause handicap (Blatteau et al., 2011).

Generally speaking, the amount of circulating mtDNA is the sum of the cell respiration and the accumulation of damage over time. Normally, and in order to deal with the breakdown of nuclear DNA, the number of copies of mtDNA is maintained (Valentin-Vega et al., 2012; Sharma et al., 2014; Itagaki et al., 2015), as is cell respiration (Ambrose et al., 2007; Patel et al., 2011), but this regulation mechanism remains blurred.

An increase in circulating mtDNA levels has been highlighted in laboratory animals which have suffered DCS (Vallée et al., 2012; Cosnard et al., 2017). The presence of extracellular DNA in the blood may be explained by necrosis (Rainer et al., 2001) or apoptosis (Jahr et al., 2001) of elements seen in the blood and vascular epithelium, or even other tissues. Studies show that 7–97% of circulating DNA would come from apoptosis (Jahr et al., 2001; Hacker et al., 2004), and this DNA would be segmented in an orderly manner into fractions with a precise size. Necrosis is normally less widespread than apoptosis and it should not be the origin of a significant amount of circulating DNA in a healthy subject. However, in certain cases it can make a substantial contribution to the increase in circulating DNA. Necrosis is induced when there are severe, irreversible lesions caused either by prolonged ischaemia, strong ionizing radiation, high temperatures, agents blocking energy production in the cells, or even agents damaging the cell membrane (Lichtenstein et al., 2001; Maas and Furie, 2009). Therefore a correlation was suggested between the amount of plasma DNA and the severity of the trauma (Lam et al., 2003). mtDNA is also released during trauma (Itagaki et al., 2015). Actually, a scenario emerges where immune cells may use mtDNA as a rapid danger messenger molecule acting in synergy with cytokines and natural antibodies (Ingelsson et al., 2018).

It is likely that when diving the quantity of circulating mtDNA corresponds to the sum of the oxidative damage and necrotic phenomena. In the case of DCS, whether expressed neurologically (central medullary or vestibular) or otherwise, necrosis seems to be the closest mechanism to what might happen during the degeneration of the vascular epithelium following abrasion by bubbles in DCS, according to the most commonly accepted theory.

Insofar as some neurodegenerative diseases have been linked to the presence of mtDNA, reactive oxygen species (ROS) and deficiency in the repair systems (Itagaki et al., 2015; Leandro et al., 2015), it is necessary to estimate the effect of the practice of diving on the circulating mtDNA levels. The aim of this work is to detect the consequences of diving and its stresses by the detection of circulating oligonucleotides of mitochondrial origin in the venous network in patients admitted to the hyperbaric center of the HIA Sainte-Anne in Toulon (France) following a diving accident. Therefore, for the first time, this work poses the question of the effect of hyperbaric exposure in humans on the mtDNA level and its place in the accident typology and its therapeutic management.

## Materials and Methods

### Subjects

In accordance with the Helsinki Declaration and the French Public Health Code, particularly its Articles in Laws L 1123-8, R 1123-32, the Southern Mediterranean Ethics Committee (14.049) and the Agence Nationale de Sécurité du Médicament et des produits de santé (French National Agency for the Safety of Medicines and Health Products) have issued a favorable opinion for the execution of this project (Ref ID-RCB 2014-A00572-45 and Ref ANSM 150112B-81), the sponsor of which is the Direction Centrale du Service de Santé des Armées (Central Directorate for the Armed Forces Health Service), with Jean-Eric Blatteau the principal investigator. The volunteers incorporated into this study have signed an informed consent form and have not received any compensation.

Recruitment took place from patients admitted to the Hôpital d’Instruction des Armées (HIA) Sainte-Anne (Toulon, France) following a diving accident. The volunteer patients presented with DCS, mostly of the neurological type (medullary, cerebral, vestibular, etc.). Subjects who experienced a procedural error during their dive (i.e., violation of decompression tables including rapid ascents, missing or interruption of stages decompression), but did not present symptoms, were also admitted as a preventive measure (Ctrl+). Healthy patients, presenting for periodic check visits, who have not dived are considered as negative controls (Ctrl-).

People who were excluded from the research were minors or people incapable of providing informed consent, patients presenting a cancer or necrotic pathology and those having practiced an intensive sport (rhabdomyolysis) in the two previous days, which could be the origin of release of oligonucleotides into the blood circulation, pregnant women even though this is a contraindication to diving, and people participating in another research study.

### Diagnosis

Diagnosis was made by hospital doctors, who are diving specialists, after questioning and clinical examination following the example of a standard care pathway. The symptoms were distributed conventionally: pain only (Type I) and serious and multi-system symptoms (Type II). Patients generally described asthenia and chest tightness with, depending on the specific nature of the accident, neurological or respiratory disorders of various types (Gempp et al., 2014). (i) Medullary decompression sickness (Medullary DCS) may be considered to be a myelopathy with focal lesions of ischemic origin, which could affect several segments of the spinal cord, and disseminated lesions caused by secondary toxic mechanisms. All this, involving paralysis in the most serious cases, particularly illustrated the course of DCS. The theory quoted most regularly for medullary type accidents is that of venous infarction, the result of the bubble generation arising in the epidural fat which embolizes the epidural veins, preferentially in the lumbar region, and thus creates ischaemia upstream. Medical imaging is rarely eloquent. It is also accepted that there is a correlation between this type of accident (Type II), i.e., serious, and the presence of a right–left cardiac shunt which encourages the passage of bubbles in the arteries. (ii) Accidents classified here as cerebro-medullary (Cerebro DCS) call to mind a clinical picture, the symptomatology of which comes from more central disorders, while considering that damage to the spinal cord may also exist. These may even lead to loss of consciousness. (iii) Vestibular decompression sickness (Vestibular DCS) is also Type II and promoted by the presence of a patent foramen ovale. It occurs mainly as harmonious rotary vertigo with nausea and vomiting. Its course is paroxysmal. It generally follows a gas embolism in one of the terminal branches of the vestibulocochlear artery, causing ischaemia upstream.

### Full Blood Analysis

Upon admission at the hyperbaric facility, venous blood samples were collected with a blood collection set (Vacutainer Brand, Becton Dickinson, Meylan, France) for blood cell counts (Sysmex xn3000 hematology analyzer, Sysmex Corporation, Kobe, Japan), biochemistry (Cobas 6000 analyzer, Roche Diagnostic Limited, Rotkreuz, Switzerland) and circulating oligonucleotides analysis (EDTA, citrate or lithium heparin tubes, BD Vacutainer, Becton Dickinson, Plymouth, United Kingdom). The blood count and blood biochemistry analyses were performed by the hospital laboratory and the data processing was carried out retrospectively.

### Circulating Oligonucleotides

Circulating oligonucleotides were quantified from blood samples (EDTA tubes: 2 ml, K2E 3.6 mg, BD Vacutainer, Becton Dickinson, Plymouth, United Kingdom) before the hyperbaric treatment. Another analysis from a subset of 22 patients was also follow-up after their hyperbaric treatment. Quantification of oligonucleotides, as previously performed (Vallée et al., 2013) in blood, requires an immediate centrifuging for 10 min at 1,500 × *g* at 4°C, and a second centrifuging of supernatants for 10 min at 20,000 × *g* at 4°C. The samples collected were stored at -80°C until analysis. 5 μl of synthetic DNA (Yakima Yellow-BHQ-1 probe, Eurogentec, Seraing, Belgium), for internal normalization, was added to the 140 μl plasma samples before extraction. DNA was extracted using a QIAcube automate (Qiagen, Venlo, Netherlands) and the NucleoSpin RNA Virus kit (Macherey-Nagel, Düren, Germany), according to the manufacturer’s instructions. Quantitative PCR was carried out with a LightCycler 480 II (LightCycler 480 Software Release; Roche Diagnostics, Mannheim, Germany) on 2 μl DNA extract added to 8 μl reaction mixture. For the no template controls, water was substituted for the extract. The reaction mixture for mtDNA amplification contained 1 μl water, 1 μl primers F and R (1 μM; Eurogentec, Seraing, Belgium), and 5 μl of an amplification mixture SyberGreen (Go Taq qPCR Master Mix 2X, Promega, Madison, WI, United States), mitochondrial primers {mitochondrial primer forward (F) [5’ ACC TCG ATG TTG GAT CAG 3’]; mitochondrial primer reverse (R) [5’ TAG ATA GAA ACC GAC CTG G 3’]}. The reaction mixture for internal control amplification contained 2 μl water, 1 μl primers delivered by the manufacturer (Eurogentec, Seraing, Belgium), and 5 μl of an amplification mixture SyberGreen (Go Taq qPCR Master Mix 2X, Promega, Madison, WI, United States). Thermocycler settings were: initialization step 94°C/10 min (denaturation step 94°C/15 s; annealing 55°C/20 s and elongation step 72°C/35 s) × 45 cycles; pre-melting 95°C/10 s, melting 55°C/10 s then up to 95°C, cooling 37°C/30 s. All assays were carried out in duplicate. Specificity was checked by melting-curve analysis, as described previously.

DNA double strand fluorometric measurements were performed on samples after both centrifugation and extraction. 4 μL of supernatant was mixed with 196 μL of a reaction mixture (QuantiFluor ONE dsDNA Dye, Promega, Madison, WI, United States), incubated according to the manufacturer’s instruction, and measured with Quantus Fluorometer (Promega, Madison, WI, United States).

### Statistics

Numerical data points are expressed as mean and standard deviation. Multiple comparisons including all groups (*n* = 114) or between divers alone (*n* = 92) were performed for each parameter using the Kruskal–Wallis (KW) test followed by the Bonferroni-Dunn *post hoc* test. The optimal cut-off level for mDNA that can discriminate between symptomatic or asymptomatic divers was determined using receiver operating characteristic (ROC) curve. The diagnostic value of this test was estimated through the calculation of sensitivity, specificity, negative predictive value and positive predictive value. With the calculation of the area under the ROC curve (AUC) and the two-tailed Fisher exact test was used to detect differences in the frequencies between symptomatic or asymptomatic animals from mtDNA threshold; odds ratio (OR) and likelihood ratio (LR) with 95% confident intervals (95% CI) were also calculated. The significance threshold was 95%.

## Results

Between 2015 and 2017, 114 volunteers (88 men and 26 women) with an average age of 45.5 ± 12.0 years (min 21 – max 74 years) took part in the study (Table 1). All were recreational divers. According to the clinical criteria there were 12 Cerebro DCS, 57 Medullary DCS, 15 Vestibular DCS, 8 Ctrl+, and 22 Ctrl-. There was no significant difference concerning age, BMI, and sex, between divers and therefore clinical status (*n* = 92, KW_age_ *p* = 0.496; KW_BMI_ *p* = 0.609, KW_sex_ *p* = 0.732). The average depths of the dives were 38.1 ± 14.9 m and an average duration of 38 ± 16 min (*n* = 92, KW_DeepmaxDCS_ *p* = 0.538; KW_TmaxDCS_ *p* = 0.609). No concomitant disease was observed by clinicians.

**Table 1:** Description of the population.

Group	*n*	Sex (% of male)	Age (years)		BMI (kg.m^-2^)		Depth max (m)		Duration (min)	
Cerebro DCS	**12**	**67%**	**44.2**		**23.6**		**43.9**		**35.8**	
				±14.0		±2.1		±21.2		±14.2
Medul DCS	**57**	**81%**	**47.7**		**22.8**		**38.4**		**38.0**	
				±11.8		±2.3		±13.7		±17.3
Vesti DCS	**15**	**73%**	**47.5**		**23.9**		**32.9**		**40.1**	
				±7.1		±2.5		±16.9		±15.3
Ctrl-	**8**	**75%**	**52.3**		**24.2**		**39.0**		**43.7**	
				±14.5		±2.5		±8.5		±12.9
Ctrl+	**22**	**77%**	**36.9**		**23.3**					
				±9.6		±1.8				


*This table resumes the main characteristics of the groups according to diagnosis group, with the sex ratio, the mean age, the mean body mass index (BMI), the maximal depth reached during the dive, and the total diving time. Data are presented as mean (bold) ± standard.*

None of the divers diagnosed with DCS stated that they had committed a procedural fault, nor a repetitive dive. All the divers had breathed a compressed air-based mixture.

### Circulating Oligonucleotides

**FIGURE 1:**
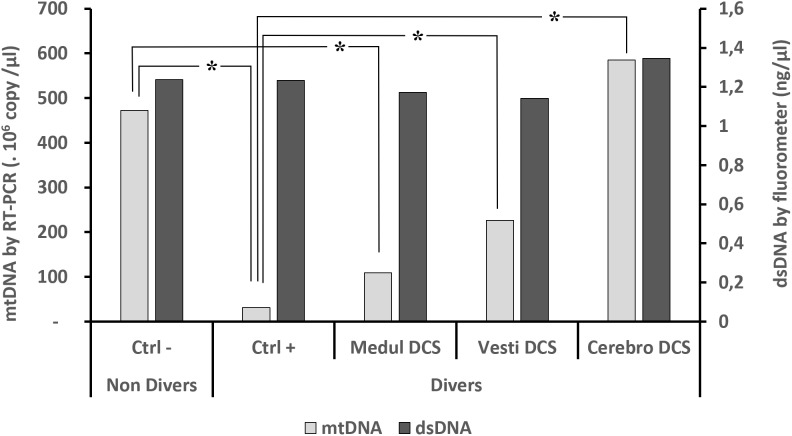
Circulating oligonucleotides according to clinical diagnosis. Quantifications of oligonucleotides were performed (1) (black histograms) by direct fluorometric measurement targeting the double strands of DNA without distinction, after the double centrifugation, and then (2) by RT-PCR (gray histograms) including the double centrifuging before the extraction/purification phase, which was followed by a qPCR targeting mDNA. Asterisk (^∗^) notes a significant difference, or μ a trend, in the KW (*n* = 114) *post hoc* analysis (*p* < 0.05).

The levels of total circulating dsDNA (Figure 1), detected in accordance with the fluorescence technique, do not show a significant difference from one group to the other, whether or not they were divers (KW_dsDNA_, *n* = 114, *p* = 0.929).

The levels of circulating mtDNA differ significantly depending on the clinical diagnosis (KW_mtDNA_, *n* = 114, *p* < 0.0001). The Ctrl- (non-divers) have significantly more mtDNA than the asymptomatic divers Ctrl+.

Compared with the Ctrl+ (asymptomatic divers), the levels of mtDNA for Vestibular DCS and Cerebro DCS are significantly higher. When reading Figure 1, overall for DCS it is seen that Ctrl+ < Medullary DCS < Vestib DCS < Cerebro DCS.

**FIGURE 2:**
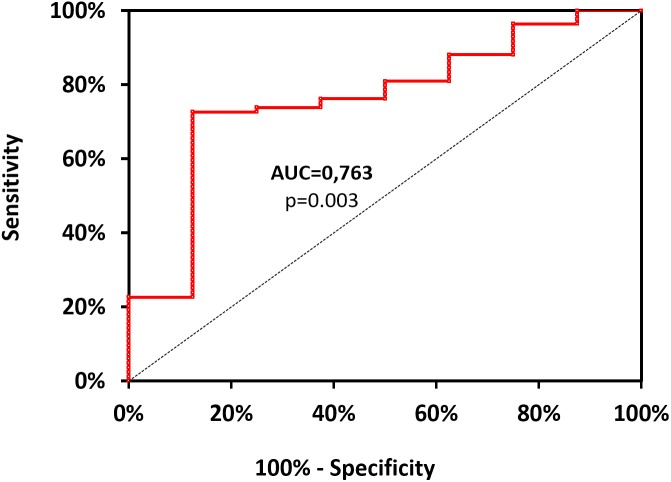
Receiver operating characteristic curve for mtDNA values. It is designed from data including lonely divers (*n* = 92), Ctrl+ being asymptomatic and therefore used as negative event. Positive events include Medullary Vestibular and Cerebro-Medullary DCS. The threshold value of mtDNA, highly predictive of clinical symptoms was determined as >16726.10^6^ copy of mtDNA/μL, with corresponding values of sensitivity and specificity as follows: 73% (95% CI, 62–81%), 87% (95% CI, 50–99%). Area under the curve (AUC), explaining differences in the frequencies between symptomatic or asymptomatic, is 0.763 (95% CI, 0.589–0.938) and its significantly different from the dotted line (*p* = 0.003).

With the use of ROC curve analysis (Figure 2) on data including divers (*n* = 92), Ctrl+ being asymptomatic and therefore used as negative event, the threshold value of mtDNA, predictive of clinical symptoms was determined as >16726.10^6^ copy of mtDNA/μL, with corresponding values of sensitivity and specificity as follows: 73% (95% CI, 62–81%), 87% (95% CI, 50–99%). Positive-predictive and value negative-predictive value are 98 and 23%, respectively. OR and LR values are as follows: OR = 18.56 (95% CI, 3.01–114.16); likelihood ratio: LR+ = 5.810 (95% CI, 0.925–36.510) and LR- = 0.313 (95% CI, 0.202–0.484). Area under the curve, explaining differences in the frequencies between symptomatic or asymptomatic, is 0.763 (95% CI, 0.589–0.938) and its significantly different from 0.5 (Fisher test: *p* = 0.003). This test failed to distinguish sick divers from healthy non-divers when including all subjects (*n* = 114, *p* > 0.05).

### Full Blood Counts

Table 2 summarizes the values for the full blood counts, the norms established by the analysis kit suppliers, and the significance levels for the inter-group differences. Only variables presenting non-compliant values or significant differences are detailed below.

**Table 2:** Full blood counts.

Variable	Mean	Cerebro DCS		Medullary DCS		Vesti DCS		Ctrl+		Normal range	KW
							
Unit	±SD									min–max	*p* =
Leukocytes		**12,975**	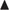	**10,193**	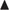	**10,170**	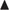	**11,195**	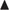	4,000–10,000	0.104
	μL		±4,720		±4,042		±3,595		±2,592		
Erythrocytes		**5.10**		**4.98**		**4.88**		**4.68**		4.00–5.40	0.620
	10^∧^6/| μL		±078		±0.45		±0.44		±0.57		
Hb		**15.5**		**15.0**		**14.8**		**14.5**		4.00–5.40	0.650
	g/dl		±2.0		±1.4		± 1.1		± 1.0		
Hematocrit		**45.0**		**43.5**		**42.8**		**42.2**		37.0–46.0	0.623
	*%*		±5.6		±4.0		±2.9		±3.0		
MCV		**88.5**		**87.5**		**87.8**		**91.0**		85–95	0.887
	fl		±4.1		±3.3		± 2.8		± 8.9		
MCH		**30.4**		**30.2**		**30.4**		**31.2**		27.0–33.0	0.979
	pg		±1.7		±1.3		± 1.1		± 2.2		
MCHC		**34.4**		**34.6**		**34.6**		**34.4**		32.0–36.0	0.835
	%		±0.6		±0.9		± 0.8		± 0.9		
Anisocytosis		**13.4**		**12.9**		**12.7**		**13.3**		11.0–15.0	**0.046**
			±0.7		±0.5		± 0.4		± 0.7		**Cerebro DCS** > **Vesti DCS**
Platelets		**253,818**		**235,491**		**247,800**		**253,750**		150,000–400,000	0.774
	/μL		±95,420		±54,334		±51,911		±41,908		
Neutrophils		**10,284**	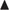	**7,865**	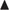	**7,960**	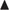	**9,466**	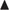	1,800–7,500	0.164
	*/*μL		±4,918		±4,069		±3,594		±2,633		
Lymphocytes		**1,830**		**1,565**		**1,482**		**972**	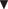	1,000–4,000	0.053
	/μL		±571		±534		±619		±239		**Ctrl+** < **Cerebro DCS**
Monocytes		**744**		**647**		**611**		**671**		200–1,000	0.530
	/μL		±253		±263		±256		±313		
Eosinophils		**86**		**90**		**79**		**39**		0–500	0.379
	/μL		±103		±80		±99		±31		
Basophils		**30**		**28**		**33**		**47**		0–150	0.255
	/μL		±24		±16		±16		±22		


*Data are presented as mean (bold) ± standard deviation according to diagnosis group. The norms (min_max) established by the analysis kit suppliers are noted in the “Range” Column, and lower or upper value are highlighted with a dark arrow. Multiple comparisons (KW column) are noted with *p*-values of Kruskal–Wallis test (*n* = 92), accompanied with their *post hoc* test translation if significant (bold).*

The average counts for circulating leukocytes are slightly raised in all divers, with a value for the Cerebro DCS of 12,975 cells per μl, for Medullary DCS 10,193, for Vestib DCS 10,170, and Ctrl+ 11,195, in comparison with the normal values established by the kit suppliers. No significant difference concerning the type of DCS was observed at this level (KW, *n* = 92, *p* = 0.104). Amongst the white blood cells, only the neutrophil population exceeds the standard values in all divers, without there being a significant difference between the groups. Without the values being able to be considered as distinctly pathological, it is however, noted that the number of circulating lymphocytes tends to be different depending on the groups (KW, *n* = 92, *p* = 0.053), particularly with low lymphocyte values in the Ctrl+. This is demonstrated by the Dunn *post hoc* tests (significance level after Bonferroni correction of *p* < 0.0083) which this time reveals significant differences between the Ctrl+ and the Cerebro DCS (*p* = 0.0058).

Furthermore, the anisocytosis values show significant differences (KW; *p* = 0.046) between the different groups of divers; however, without these values exceeding the normal ranges and being considered pathological. Thus, the *post hoc* tests demonstrate a tendency toward a difference between the Cerebro DCS and the Vestib DCS (*p* = 0.0089), allowing the prediction of a difference in the distribution of red blood cell sizes (It should be remembered that reticulocytes are slightly larger than mature erythrocytes so that, overall, a raised MCV may be due to a large number of these immature cells). However, no significant difference was observed with regard to the Mean Corpuscular Volume: the average size of the red blood cells.

### Blood Biochemistry

Table 3 summarizes the values for major indices in haemostasis, the norms established by the analysis kit suppliers, and the significance levels for the inter-group differences. Only variables presenting non-compliant values or significant differences are detailed below.

**Table 3:** Major indices in hemostasis.

Variable	Mean	Cerebro DCS		Medullary DCS		Vesti DCS		Ctrl+		Normal range	KW
							
Unit	±SD									min–max	*p* =
Platelets		**253,818**		**235,491**		**247,800**		**253,750**		150,000–400,000	0.774
	/μL		±95,420		±54,334		±51,911		±41,908		
Quick time		**13.5**		**13.2**		**13.5**		**13.2**			**0.454**
	sec		±0.9		±0.6		±0.7		±0.9		
Prothrombine		**94.2**		**97.1**		**96.3**		**95.3**			0.901
	%		±8.1		±4.3		±6.4		±9.5		
APTT ratio		**1.0**		**1.0**		**1.0**		**1.0**		<1.20	**0.830**
			±0.1		±0.1		±0.1		±0.1		
Fibrinogen		**3.24**		**2.99**		**2.77**		**3.52**		2.00–4.00	0.073
	g/l		±0.85		±0.61		±0.39		±0.35		Ctrl+ > Vesti DCS
D Dimers		**1.04**	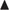	**0.51**		**0.35**		**0.39**		0.00–0.50	0.092
	/μg/ml		±0.90		±0.49		±0.11		±0.17		Cerebro DCS > Med DCS


*Data are presented as mean (bold) ± standard deviation according to diagnosis group. The norms (min_max) established by the analysis kit suppliers are noted in the “Range” Column, and lower or upper value are highlighted with a dark arrow. Multiple comparisons (KW column) are noted with *p*-values of Kruskal–Wallis test (*n* = 92), accompanied with their *post hoc* test translation if significant (bold).*

Averages for the raised levels of D-Dimers (μg/ml) (fragments from the breakdown of fibrin to monitor hypercoagulability) have been recorded in the Cerebro DCS (1.04), and the Medullary DCS (0.51), in comparison with the expected normal values of between 0.00 and 0.50. It seems a tendency to be different between DCS type is emerging (KW: *n* = 92, *p* = 0.073), particularly suggesting that the level of Cerebro DCS would be greater than that of Medullary DCS (*post hoc* test: *p* = 0.012) (Table 3).

The fibrinogen averages tend to be different (KW; *n* = 92, *p* = 0.073), without it being possible to present the values as pathological. The Ctrl+ values tended to be higher than those of the Vestib DCS (*post hoc* test: *p* = 0.013) (Table 3).

Table 4 summarizes the values for blood biochemistry, the norms established by the analysis kit suppliers, and the significance levels for the inter-group differences. Only variables presenting non-compliant values or significant differences are detailed below.

**Table 4:** Blood biochemistry.

Variable	Mean	Cerebro DCS		Medullary DCS		Vesti DCS		Ctrl+		Normal range	KW
							
Unit	±SD									min–max	*p* =
Sodium		**139**		**138**		**139**		**141**		136–145	0.107
	mmol/l		±3		±**2**		±2		±3		
Potassium		**4.1**		**4.1**		**4.1**		**4.3**		3.5–5.1	0.780
	mmol/l		±**0.3**		±0.4		±0.3		±0.6		
Chlorures		**102**		**101**		**102**		**103**		98–107	0.187
	mmol/l		±3		±**3**		±2		±1		
CO_2_ total		**26.1**		**27.0**		**25.9**		**26.0**		22.0–29.0	0.592
	mmol/l		±3.4		±2.6		±2.5		±2.3		
Anionic gap		**15.7**		**14.8**		**15.9**		**16.8**	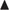	8.0–16.0	0.372
	mmol/l		±3.2		±3.0		±1.9		±3.0		
Protein		**69.1**		**70.9**		**70.2**		**72.0**		64.0–83.0	0.321
	g/l		±6.3		±5.4		±4.0		±2.8		
Glycemia		**6.9**	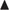	**5.9**		6.0		**6.0**		3.9–6.1	0.345
	g/l		±2.2		±1.6		±0.8		±2.0		
Creatinine		**80.8**	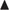	80.8	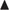	**72.9**		**83.5**	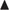	44–80	0.369
	μmol/l		±**16.1**		±15.6		±11.2		±22.1		
GFR MDRD		**88.4**	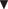	86.6	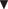	**96.0**		**74.8**	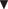	>90.0	**0.033**
	ml/mn/l,73 m^3^		±19.0		±14.2		±**14.1**		±9.9		**Ctrl+ < Vesti DCS**
GFR CKD-EPI H creat		**92**		**97**		**101**		**87**	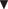	>90.0	0.254
	ml/mn/l,73 m^3^		±18		±12		±10		±11		
Uric acid		**345**	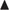	**320**		**286**		**398**	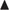	143–340	**0.037**
	μmol/1		±134		±75		±56		±88		
Cholesterol		**5.02**		**5.34**	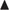	**5.44**	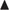	**5.33**	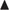	3.00–5.20	0.911
	mmol/l		±**1.40**		±1.20		±1.09		±1.26		
HDL-c		**1.93**	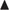	**1.74**	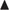	**1.80**	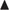	**2.08**	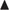	1.04–1.50	0.801
	mmol/l		±**0.53**		±0.47		= 0.61		±1.11		
LDL-c		**5.92**	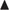	**3.07**		**3.14**		**2.85**		1.75–3.40	0.874
	mmol/l		±8.14		±0.92		±1.02		±**0.54**		
TGO (ASAT)		**25**		**23**		**23**		**21**		11–32	0.707
	Ul/I		±**6**		±**6**		±6		±3		
TGP (ALAT)		**24**		**21**		**17**		**20**		12–66	0.408
	Ul/I		±12		±11		±4		±12		
GGT		**55**		**29**		**19**		**21**		11–71	0.065
	Ul/I		±76		±20		±11		±10		Cerebro DCS > Vesti DCS
CK		**234**	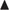	**213**	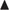	190		**210**	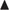	26–192	0.199
	Ul/I		±111		±127		±195		±101		
CRP		**7.5**	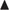	**2.5**		**1.9**		**2.1**		<5.0	0.144
	mg/l		±16.3		±4.2		±3.0		±1.4		
Albumin		**41.6**		**41.6**		**43.8**		**43.6**		35.0–52.0	0.521
	g/l		±3.8		±4.6		±4.2		±1.9		


*Data are presented as mean (bold) ± standard deviation according to diagnosis group. The norms (min_max) established by the analysis kit suppliers are noted in the “Range” Column, and lower or upper value are highlighted with a dark arrow. Multiple comparisons (KW column) are noted with *p*-values of Kruskal–Wallis test (*n* = 92), accompanied with their *post hoc* test translation if significant (bold).*

The average values of levels concerning the anion gap are slightly raised in the Ctrl+ (16.8), in comparison with the normal values of between 8.0 and 16.0 mmol/l. A high anion gap would indicate the presence of an excess of anions that were not assayed (lactates, phosphates, sulfates, plasma proteins) and therefore metabolic acidosis.

The average values for glycaemia are high in the Cerebro DCS (6.9), for expected values of between 3.9 and 6.1 mmol/l.

The average values for creatinine are high in the Cerebro DCS (80.8), the Medullary DCS (80.8) and the Ctrl+ (83.5) for normal values of between 44.0 and 80.0 μmol/l.

The average values for the glomerular filtration rate GFR MDRD (ml/mn/1.73 m^3^) are low in the DCS (88.4), the Medullary DCS (86.6), and the Ctrl+ (74.8), for expected values that are normally higher than 90. A significant difference emerges (KW; *p* = 0.033), particularly between the Ctrl+ for which the filtration rate remains low compared with the Vestib DCS (Dunn *post hoc* test, *p* = 0.006). The GFR may, however, be assessed by the other glomerular filtration rate calculation method (CKD-EPI H Creat) where the Cerebro DCS (91.8), the Medullary DCS (97.2), the Vestib DCS (100.8) and the Ctrl+ (87.3) have slightly higher average values, thus putting forward the idea of renal failure, possibly moderate. This time no significant difference was observed.

The average values for uric acid are high in the Cerebro DCS (345 μmol/l) and the Ctrl+ (398 μmol/l) compared with the normal expected values of between 143 and 340 μmol/l. A significant difference emerges (KW; *n* = 92, *p* = 0.037), without showing an inter-group difference by the *post hoc* tests.

The average values for total cholesterol are high in the Medullary DCS (5.34) Vestib DCS (5.44) and Ctrl+ (5.33) for normal expected values of between 3.00 and 5.20 mmol/l. There is an excess of HDL-c (high density lipoprotein-cholesterol) in all the divers [Cerebro DCS (1.93), Medullary DCS (1.74), Vestib DCS (1.80) et Ctrl+ (2.08); expected values of between 1.04 and 1.50 mmol/l]. Only the Cerebro DCS have an average value for LDL-c higher (5.92) than the norm (1.75–3.40 mmol/l).

The averages for GGT tend to be different (KW; *p* = 0.065), particularly between the Vestib DCS and the Cerebro DCS (*post hoc* test: *p* = 0.010), without it being possible to present the values as pathological.

The average values for creatinine kinase are high in the Cerebro DCS (234), the Medullary DCS (213) and the Ctrl+ (210); normal values of between 26 and 192 IU/l.

The averages for CRP (C-reactive protein) are high in all the Cerebro DCS (7.5) for normal expected values of below 5.0 mg/l.

### Effect of Hyperbaric Oxygen

In the context of their continuity of care, 26 patients were sampled. This made it possible to quantify the circulating mtDNA just after their treatment table. Out of 22 Medullary DCS, 14 were treated with the B18 table and 8 with the modified Comex 30 table (Cx30). The 2 Cerebro DCS, like the 2 Vestib DCS, were each treated with a different table. Only the Medullary DCS were analyzed for homogeneity reasons. The Ctrl– experienced an A15 air table.

The hyperoxic hyperbaric treatments were either of the B18 type (150 min, maximum pressure 2.8 ATA, 370 OTU, 235% CNS toxicity), or the modified Comex 30 type (330 min, maximum pressure 4.0 ATA, 792 OTU, 555% CNS toxicity), or the A15 type (50 min, maximum pressure 2.5 ATA, 166 OTU, 112% CNS toxicity). The B18 and Cx30 tables experience air or pure oxygen intermittently, with in addition a Nitrox 50 mixture from the start for the modified Comex 30 table. The Comex 30 table is usually reserved for the most severely afflicted patients.

**FIGURE 3:**
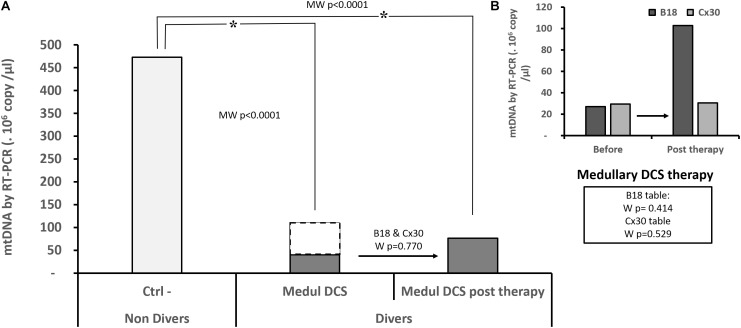
Quantity of circulating mitochondrial DNA in the patients suffering from medullary DCS **(A)**, before and after treatment by B18 (*n* = 14) or Cx30 (*n* = 8) **(B)**. Only Medullary DCS patients with values before and after treatment are compared to the non-diving subject (Ctrl**-**, *n* = 22): the dots represent the totality of medullary DCS patients (*n* = 57) **(A)**. Asterisk (^∗^) means that the statistical difference is significant. KW *p* < 0.0001 *post hoc* Conover-Iman with a corrected significance level (Bonferroni) of 0.0167. NS shows an absence of statistic difference for the paired samples (Wilcoxon test: W).

More specifically concerning the Medullary DCS whom it has been possible to follow-up (Figure 3), their level of mtDNA remains significantly different from the Ctrl- subjects who have not dived (KW_Ctrl-/MedulDCS/MedulDCSpost-therapy_, *p* < 0.0001). The analysis of paired data has not made it possible to show an evolution in their DNA level after treatment, whether the tables are considered together (W_B18andCx30_, *n* = 22, *p* = 0.770) or separately (W_B18_, *n* = 14, *p* = 0.414; W_Cx30_, *n* = 8, *p* = 0.529).

## Discussion

### Effect of Diving on mtDNA Levels

The stress imposed by diving is reflected particularly in the cells via the measurement of circulating mitochondrial oligonucleotides, the quantity of which is higher in non-diving subjects (Ctrl-) than in healthy divers (Ctrl+). This result spotlights an effect specific to diving, and more especially of hyperbarism, as the sample was realized upon the admission at the hyperbaric facility and before the hyperbaric treatment: this result is comparable to the result we have described in animals (Cosnard et al., 2017).

### Effect of Diving Accidents on mtDNA Levels

For DCS, the increase in circulating mitochondrial oligonucleotides in laboratory animals was basically attributed to cell destruction by bubble abrasion (Vallée et al., 2013). The release of mtDNA by the platelets may also be mentioned (Boudreau et al., 2014) if their activation during DCS is considered. The comparison of the results of this study in humans supports this theory about DNA, because taken overall healthy divers have less mtDNA than subjects with DCS. The analysis of the ROC curve suggests that an increase of mDNA level in the plasma (>16726.10^6^ copy of mtDNA/μL) of a diver is significantly predictive of symptoms occurrence related to DCS. Nonetheless, it should be noted this test failed to differentiate sick divers from healthy non-divers, as a result of which it should only be use for divers. However, these results also add the concept of temporality and additive effect insofar as the fact of diving induces an initial reduction in circulating mtDNA levels – possibly attributable to oxidative stress – and where the necrosis induced later by the presence of circulating bubbles would release them again. In other words, the level of stress when diving -whatever it is- would become a predisposition to diving accidents, not necessarily enough but encouraging the rupture of cell integrity by the bubbles (DCS).

The study published by Mehta et al. (2017) therefore notes that the increase in circulating mtDNA would be associated with neuro-inflammation and the reduction in circulating mtDNA to a long-term neurocognitive deficiency. The Cerebro DCS would therefore be in an acute neuro-inflammatory phase insofar as the mtDNA levels are much higher than in the asymptomatic divers (Ctrl+), and the neutrophil levels for CRP and D-Dimers exceed their usual values. This would also amount to the interpretation that the Vestib DCS and Medullary DCS would experience less pronounced neuro-inflammation. While it does not seem very reasonable to assume that the Ctrl+ divers present a neurocognitive deficiency at the time, the Hemelryck’s study using neuropsychometric tests concluded that accident-free divers may have some long-term adverse effects on short-term memory (Hemelryck et al., 2014). However, this study did not show evidence of general higher cognitive function deficiency. A subsequent study could be advantageous to deal with this point.

### Effect of Diving and Accidents on dsDNA Levels

It seems counter-intuitive that the amount of dsDNA does not change across samples with or without an acute (serious) illness. Intuitively the cell damage associated with the mechanical stress at vascular level due to micro- and macro-bubbles, the increased oxidative stress, and immune reaction associated with the thromboembolic event should lead to an increase of the total dsDNA, at least in DCS compared with negative controls. Indeed, independently from mtDNA variation, if at the base of the its release, in diseased patients, there is a cell damage, then the nuclear DNA should be released as well. However, this study does not highlight any difference between divers and non-divers for the total DNA. This leads us to suppose _1st _ total DNA is not an efficient marker of DCS, and/or _2nd _ cell destruction, by shear stress, is possibly not the major phenomena in DCS. We also discussed an analog result in our animal model of DCS, where it was concluded that an increase in circulating dsDNA could be related to the presence of bubble, while a decrease in mtDNA should be specifically linked to the effect of the hyperbaric exposure (Cosnard et al., 2017).

### Full Blood Counts in Diving Accidents

In all the divers our results show an immune reaction via the mobilization of leukocytes and more especially neutrophils, and also CRP in the Cerebro DCS. The mtDNA results in leukocyte activation (Boudreau et al., 2014; Perez-Santiago et al., 2017), particularly activation of neutrophils (Zhang et al., 2010a; Itagaki et al., 2015). The neutrophils are also likely to release reactive oxygen intermediaries (ROS) from their secondary granules and proteases. Our results would seem to be compliant with the documentation, insofar as mtDNA has many CpG motifs, inherited from the endosymbiotic origins of mitochondria. These motifs label the circulating mtDNA as a “damage-associated molecular pattern” molecule (DAMP), which activates inflammation via the toll-like receptors (TLR)-9 (West et al., 2006; Walko et al., 2014; Timmermans et al., 2017) to result amongst other things in the formation of extracellular traps (Itagaki et al., 2015). The TLR-9s are especially involved in the innate immunity and recruitment of neutrophils (DeFranco et al., 2009). Their polymorphism causes exposure to hepatic, (Kiziltas, 2016) renal (Yang et al., 2013), or thrombotic disorders (Morgand et al., 2014), atopic eczema (Novak et al., 2007), osteoarthritis (Balbaloglu et al., 2017) or even multi-organ failure (Zhao et al., 2012). Cell destruction following trauma may release mtDNA which is the origin of systemic auto-inflammation by neutrophils mediated by the TLR-9s (Sibilia, 2007; Zhang et al., 2010a; Itagaki et al., 2015).

### Blood Biochemistry

#### Global Effect of Diving and Accidents

In all the divers, the analysis of the blood biochemistry results highlights high levels of HDL-c, creatinine, and creatinine kinase, in addition to low glomerular filtration rates.

The low glomerular filtration rates may be explained by the diver being dehydrated, a result of the “blood shift” resulting from the immersion of his/her body, itself being the origin of an increase in renal function and extravasation (Jimenez et al., 2010; Castagna et al., 2013). It should be noted that the reduction in rates may continue despite the intravenous fluids administered during the initial medical treatment (before arrival at the hyperbaric center). Renal failure may also be suspected but this is not described in the literature relating to diving.

The HDL-c values are shown to be higher than usual in all the divers. Without neglecting the impact that the (moderate) age of the divers can have on this variable, this would indicate either the impossibility of the HDL lipoproteins recovering (transporting) cholesterol in the tissues to take it to the liver, or a reduction in their absorption by hepatocytes. Normally a high level of HDL would protect against infarctions (Blann and Ahmed, 2014). Effectively, in normal conditions the HDL prevents oxidation of LDL-c, whereas the HDL-c undergoes structural changes in pathological conditions such as oxidative stress, inflammation and diabetes, which reduces the anti-arteriosclerotic and anti-inflammatory functions (Ahn and Kim, 2016). The high levels of HDL-c (for all divers) recorded in this study would, therefore, be the result of hyperbaric/hyperoxic exposure rather than the only consequence of bubble genesis induced by the decompression. It should be noted that the liver may contribute to the amplification of the inflammation but also suffer from it via the stimulation of its TLRs (Kiziltas, 2016), especially by the binding of the mtDNA to its TLR-9s (Zhang et al., 2010a,b).

In this study, the LDL-c (low density lipoprotein-cholesterol) is only raised in the cerebro-medullary DCS. The LDL lipoproteins carry cholesterol from the liver to the cells. The LDL may be modified by oxidation or glycation, and so be recognized by non-specific receptors (such as the CD36 co-receptor of TLR2+TLR6) on the surface of hepatocytes, macrophages or other cells and thus participate in inflammation (Blann and Ahmed, 2014). A high level of LDL-c would be seen by the inability of the liver to bind it.

In addition, the high creatinine values, resulting from protein breakdown or renal failure, in all the divers, must also contribute to placing the liver’s purification functions under massive stress.

When combined with a high cholesterol level as in the Medullary and Vestibular DCS and even the Ctrl+, this would result in deregulation of the hepatic system and cell stress. Effectively, the creatinine kinase levels are high or slightly high in all the divers, which heralds a mild cell stress.

Finally, and without being significant, the limitation of the body’s purifying functions would increase the risk of auto-immune reaction by the circulating mtDNA (Ingelsson et al., 2018).

#### Specific Characteristics of Clinical Cases

Beyond the biochemical changes previously specified, the Cerebro DCS present an inflammatory status probably linked to a thrombotic status demonstrated by the high values of D-Dimers. The bubble-platelet interaction is regularly spotlighted in DCS. However, beyond its well-known thrombotic role, it would also be interesting to consider the involvement of the platelets in the immune response (Harifi and Sibilia, 2016).

Without being excessive the hyperglycemia recorded in the Cerebro DCS heralds moderate systemic metabolic failure with an inability of the cell to use or store glucose, even more specifically a failure of the respiratory chain.

In the Cerebro DCS an increase in waste from protein breakdown (degradation of DNA purines, hypoxia, cytolysis) is noted more specifically. This is shown by the increase in uric acid. In addition, the LDL-c is raised, like the other hepatic markers described, which leaves us to suppose either that the liver is moderately affected in line with an inflammatory syndrome (neutrophils and CRP) or the maximum level of liver purification is affected.

### Perspective

The increase in hydrostatic pressure implies that the diver is hyperoxic for air diving (400 mbars of O_2_ at a depth of 10 m, 600 mbars at a depth of 20 m, etc.) and even more so with dives using oxygen-enriched mixtures of the Nitrox type (nitrox 32: 960 mbars once a depth of 20 m is reached), even if saturation is incomplete. Paradoxically hyperoxic exposures can inhibit aerobic respiration by overcrowding of the ROS around the mitochondria (Resseguie et al., 2015) and thus promote the anaerobic chain. It has regularly been possible to demonstrate oxidative stress in diving by describing, for example, an overproduction of ROS or HSP (heat shock protein) (Eftedal et al., 2013) in humans. For example, in the rat the effects of hyperbaric oxygen change the dopaminergic neurotransmission of the basal ganglia, an essential structure for controlling extrapyramidal motor function from 300 mbars (Adachi et al., 2001), and persistent reduction of dopamine levels is seen from 1,000 mbars (Lavoute et al., 2014). Oxidative stress and cell damage occur when the equilibrium between the production of ROS and the cell’s detoxification capacity is broken (O’Reilly, 2001). It targets the guardians of the genome and, more especially, the repeated sequences rich in guanine from telomeres (Thilagavathi et al., 2013a,b; Mishra et al., 2016). It affects the integrity of the DNA, particularly the mtDNA which, due to its lack of protection by histones and a limited repair system, is more subject to damage induced by oxygen than nuclear DNA (Wallace et al., 1995; Copeland et al., 2002). Its breakdown contributes to cell respiration dysfunction and then probably to anticipated cell death (Shokolenko et al., 2009; Resseguie et al., 2015; Hobani, 2016; Rima et al., 2016). In the cells of mammals the oxygen toxicity is seen as a breakdown of DNA after several minutes’ exposure as soon as the quantity of oxygen doubles (200 mbars) (Cantoni et al., 1989; Cacciuttolo et al., 1993), with, it seems, better resistance in the cells of people already exposed to high oxygen levels (Witte et al., 2014).

In this context, the measurement of the mtDNA levels seems relevant in the patient with DCS presenting neurological symptoms. Effectively, neurological pathologies only require a small lesional extent to generate significant symptoms, amongst other reasons because the nerve tissues, with their high energy requirements, have more copies of mtDNA than other tissues (Xie et al., 2015). In addition, the tissues of the central nervous system (CNS) present high levels of phospholipids, such as arachidonic acid, docosahexaenoic acid, inositol phosphate or even diacylglycerol, which are all easily peroxidized thus generating ROS. Normally these phospholipids serve as secondary messengers and contribute to the correct functioning of the CNS (Mahadik and Evans, 2003). Furthermore, neurological DCS is rarely due to an embolization (Yanagawa et al., 2016). To support this hypothesis, there is also regular absence of trauma signs visible on imaging and the not insignificant possibility that this picture worsens during hyperbaric oxygen therapy, which generates additional ROS.

The quantity of circulating mitochondrial oligonucleotides is higher in non-diving subjects (Ctrl-) than in healthy divers (Ctrl+) and this is comparable to the result we have described in animals (Cosnard et al., 2017). This former study, where the maximum partial oxygen pressure reaches more than 2100 mbars, has shown that rats which have not suffered DCS following a hyperbaric protocol have lower mtDNA levels than those which have not dived (Cosnard et al., 2017). To our knowledge, there is no description of the effect of pressure *per se* or of gases diluting the oxygen (nitrogen or helium) on the integrity of genome components (Brubakk et al., 2003b) for the pressure values mentioned in this study. By default, it is possible to suggest that the reduction in mtDNA levels could be the result of oxidative stress. We have evaluated the oxygen contents for this study and discussed their consequences in the section “Supplementary Materials.” When diving, the most severe limitations are imposed by neurological poisoning with oxygen, because the relatively high susceptibility of the brain tissues makes them likely to be affected by lower oxygen tensions than most other organs (Brubakk et al., 2003b). Also, the only hypothesis that we can put forward at this stage would be mitochondrial deregulation linked to oxidative stress (Shokolenko et al., 2009; Resseguie et al., 2015) causing breakage to the strands of amplified sequences linked to hyperbaric exposure and its high oxygen contents. In particular, this hypothesis is based on the overproduction of ROS and HSP well described in diving (Eftedal et al., 2013) and the impact of these components on the integrity of DNA strands (Shokolenko et al., 2009; Hobani, 2016; Rima et al., 2016). It is also based on the work of Cacciuttolo et al. (1993) and Witte et al. (2014) describing a direct effect of hyperoxia on the degradation of DNA strands in mammal cells. In addition, no variation in double strand DNA levels are noted in this study. They are measured by a non-specific technique of one sequence and where a breakage cannot interfere with the measurement (see also Cosnard et al., 2017).

When considering the previously quoted work, it is necessary to pose the question of the toxicity of hyperoxic hyperbaric and normobaric exposures in humans, particularly when they are conducted for therapeutic purposes. mtDNA is highly sensitive to oxidation and many studies suggest that a reduction in mitochondrial function and/or oxidative stress would be the origin of neurodegenerative diseases (Coskun et al., 2012; Ganguly et al., 2017; Mehta et al., 2017; Rajda et al., 2017). Effectively, the circulating mtDNA levels are lower in patients suffering from Alzheimer’s diseases or Parkinson’s disease compared with healthy subjects (Podlesniy et al., 2013; Pyle et al., 2015). As well as the production of ATP, the mitochondria are responsible for calcium homeostasis, a not insignificant part of the innate immune response and of the cell death program (West et al., 2011).

Although it has been conducted in animal study (Vallée et al., 2013), the lack of imaging for bubbles should also be mentioned as a limitation and, it could be interesting to investigate it in future studies with regards to the results highlighted here. It could also be discussed as limitations that – (a) diving could be perceived as exercise _and this last should influence the levels of mtDNA detected_ (b) raising the problem of the choice of the controls chosen and their absence of diving/exercise. Nonetheless, the same study implying animals did not required exercise and the results were similar to those presented here (Vallée et al., 2013).

The difference between the number of subjects in each category could influence the lack of significant differences found and it may very well be that larger samples would conduct to significant differences.

Finally, we could regret that no measurement of oxidative stress could have been performed in patients of this study. Nonetheless, this stress is sufficiently well described in literature to consider it in this study. This pathway is worthy of serious exploration, considering therefore the very recent publication of McEvoy (2018) wondering if using oxygen in excess is not just “adding fuel to the fire.”

### Reconsiderations, Moderation, and Limitations

#### Reconsideration of Previous Works

At the same time, recent studies conducted in animals have suggested that fluoxetine, an antidepressant, has an anti-inflammatory effect, which works by suppressing oxidative stress (Caiaffo et al., 2016) particularly induced by the SERT approach/p38 MAPK/Nrf2 (Wang et al., 2017). The most recent literature describes fluoxetine as inhibiting oxygen consumption and reducing ATP synthesis (Charles et al., 2017; Elmorsy et al., 2017; Villa et al., 2017). This result corroborates our studies conducted previously in animals where the incidence of neurological DCS is largely reduced by fluoxetine (Blatteau et al., 2012, 2015; Vallée et al., 2016). The levels of transthyretin, a molecule synthesized by the liver, reduced in animals declaring a DCS but its levels did not seem to vary in the healthy human diver (Lautridou et al., 2017). It is also possible to comprehend the results concerning the involvement of the TREK-1 channels in DCS (Vallée et al., 2012, 2016) by looking at it again and highlighting the increased susceptibility of the TREK-1^-/-^ animals linked to their sensitivity to hyperoxia (Schwingshackl et al., 2014).

Finally, and considering the effects described here of the mtDNA and other inflammatory markers, whilst relying on the work of Sibilia (2007), it would seem that DCS may be qualified as an auto-inflammatory disorder – it continues despite the disappearance of the sterile bubbles, the air in the bubbles not strictly speaking being a pathogenic and neutrophilic element (Thom et al., 2015). In which case it would seem interesting to research the genetic or epigenetic predispositions favoring DCS.

#### Limitations and Moderation

**FIGURE 4:**
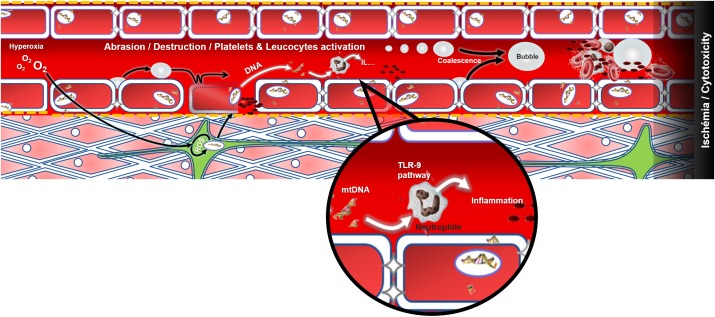
Modeling of decompression sickness incorporating the genesis and increase of bubbles at the vascular level. Excess oxygen could be the source of oxidative stress which alters mitochondrial function and weakens the vascular (red), neuronal (green) or muscular (pink) tissues for example. Following decompression, the bubbles may either abrade the endothelium and leave the collagen bare (yellow dots) which will activate the platelets (in black), or activate the platelets and the immune system directly when they make contact, or even cause an obstruction to the circulation. A dark area is established in the end downstream of the thrombosis: this last illustrating the sum of effects, like cytotoxicity and ischemia among athers, that may spread in the body and contribute to the vicious circle of DCS which is poorly understood. The release of mtDNA would be concomitant and would also participate in the activation of the immune system.

Patients arrive at different times after the dive but overall they present themselves at the onset of the disease (except for the Ctrl+). It is also necessary to take into account the environmental context and the various inter-individual susceptibilities of humans, and therefore consider the possibility of profiles that are particularly sensitive to hyperoxia. The possibility of confounding factors escaping the study context must also be considered. Asthenia, paresthesia or the feeling of chest tightness are as attributable to psychological stress as they are to physical or biochemical stress. So a simple mitochondrial fault inducing hypoxia, and resulting from oxidative stress during the dive, may have equal central, medullary or vestibular repercussions, provided that a localized anatomical fragility is expressed in the patient. In addition, symptoms are not exclusive and overlap. The various components of DCS compete in the realization and maintenance of a multi-factorial ischaemia where the cytotoxic phenomena overlap little by little with the effects of tissue oxygen deficit (Figure 4).

The initial therapeutic management could interfere with this analysis. Before the blood sample can be taken it involves possible medication with aspirin (taken on the boat as self-medication), and possible plasma expansion (1 L of water in 30 min on the boat or an intravenous infusion by the Emergency Services). To our knowledge, no studies on these substances have made a direct link with the circulating mtDNA levels, even if it is evident that they have pharmacological properties. The effects of aspirin are discussed (Montcalm-Smith et al., 2008; Bessereau and Annane, 2013; Westerweel et al., 2013). Aspirin is used as an anti-aggregating agent but the average number of circulating platelets remains unchanged in this study. It had no effect in animals (Pontier et al., 2011; Lambrechts et al., 2015) even though platelet aggregation was confirmed. Recent publications describe aspirin as limiting the normal flows of ions and metabolites. This disturbs the cell calcium homeostasis and the potential of the mitochondrial membrane by closing the voltage-dependent ion channels 1, and which could finally be the origin of apoptosis, linked to oxidative stress that could be prevented by the use of *N*-acetyl cysteine (Raza et al., 2016; Tewari et al., 2017). Interestingly, combining it with bacterial lipopolysaccharides potentializes its toxicity (Raza et al., 2016).

The hyperbaric center uses hyperoxic hyperbaric treatment spread over one or more sessions. Only the first session is considered in this study. The hyperoxic hyperbaric treatments were either of the B18 type (150 min, maximum pressure 2.8ATA, PiO_2_max = 2800 mbars, 270 OTU, 235% CNS toxicity), or modified Comex 30 type (330 min, maximum pressure 4.0 ATA, PiO_2_max = 2800 bars, 792 OTU, 555% CNS toxicity). These two tables experience air or pure oxygen intermittently, with in addition a Nitrox 50 mixture from the start for the modified Comex 30 table. The Comex 30 table is usually reserved for severely afflicted patients. Only the Medullary DCS who could be followed-up were analyzed. Whichever table is considered, the analysis of their mtDNA has not made it possible to demonstrate an evolution of their mtDNA levels after the therapeutic treatment. In the end these remain low. With regard to the arguments quoted earlier, it is probable that the oxidative stress continues, the follow-up of patients who were not treated, or healthy people newly exposed, could in fact confirm this hypothesis.

The provision of oxygen therapy should not be denied, particularly by the nature of the pre-conditioning (Gempp and Blatteau, 2010), or simply by the necessity of ROS in physiological quantities to initiate the essential immune functions, whether they are antiviral, antibacterial or anti-parasitic (West et al., 2011; Sena et al., 2013). Its use is also recognized in limiting apoptosis and promoting cell regeneration in the renal tubules, for example (Migita et al., 2016).

## Conclusion

This work demonstrates that accident-free divers have less circulating mtDNA than non-divers, which leads to the assumption that hyperbaric exposure degrades the mtDNA. mtDNA levels is on average greater in divers with DCS compared with accident-free divers. On another hand, the amount of dsDNA does not change across samples with or without a DCS. Initially the increase in circulating oligonucleotides was attributed to the destruction of cells by bubble abrasion following necrotic phenomena. If there really is a significant difference between the levels of mtDNA for the Ctrl- and the Ctrl+ or the Medullary DCS suggesting the effect of oxidative stress, this difference is not significant between these same DCS and the Ctrl+. This refutes the idea of massive abrasion linked to the presence of bubbles and would suggest that oxidative stress could be a key element without necessarily being sufficient, for the occurrence of neurological type damage.

## Author Contributions

J-EB and NV conceived and designed the research. SG and NV performed the analysis. J-JR and NV analyzed the data. J-EB, SG, SR, SDM, J-JR, and NV interpreted the results. NV prepared the figures. J-JR and NV drafted, edited, and revised the manuscript. J-EB, SG, SR, SDM, PL, EG, AD, HL, JM, OC, JA, J-JR, and NV approved the final version of the manuscript.

## Conflict of Interest Statement

The authors declare that the research was conducted in the absence of any commercial or financial relationships that could be construed as a potential conflict of interest.
