# Supplementary material for: Reduction in the Level of Plasma Mitochondrial DNA in Human Diving, Followed by an Increase in the Event of an Accident
Source: Front Physiol. 2018 Nov 29;9:1695. doi: 10.3389/fphys.2018.01695 (PMC6282000; doi:10.3389/fphys.2018.01695)
Supplement: Supplementary file 1 [file Data_Sheet_1.pdf]

Supplementary Data 1

For this study and at least considering that the gas inhaled was air, we assess the maximum partial pressure of oxygen inhaled at 1010±523 mbars (depending on the average maximum depth 38.1±14.9 m) for decompression accidents (38.0±15.5 m i.e.P_iO2=1008±535 mbars) and procedural errors (39.0±8.5m i.e. P_iO2=1029±388 mbars). When diving the maximum partial pressure of diatomic oxygen breathed by the diver is regulated to 1600 mbars internationally. However, the recommendations of the FFESSM (France) are a maximum Pi02 of 1400 mbars, and the Royal Netherlands Navy considers that oxygen toxicity may come into play at 1300 mbars (Wingelaar et al., 2017). Considering the empirical calculation of the OTUs (Oxygen Toxicity Unit) for pulmonary toxicity (Hamilton Jr, 1997), the diver would potentially have 312 cumulative diving minutes at a Pi 0_2 of 1600 mbars before reaching the value of 600 OTU. This remains far from the legal accepted dose of 1500 OTU by the Anglo-Saxons (REPEX Table), and would a posteriori authorise hyperbaric treatment with oxygen (a B18 table represents 370 OTU and a Cx30 555 OTU). The value of 615 OTU would induce a reduction in the vital respiratory capacity of 4% (Hamilton Jr, 1997). If it seems that the lungs can be partially spared – studies show an increase in alveo-capillary patency due to oxygen (Brubakk et al., 2003) - it would not be the same for the central nervous system. In fact, using these considerations, groups of divers have been exposed to repetitive doses of oxygen that are tolerable for the lungs, but which have finally triggered asthenia and paraesthesia, well before reaching the supposed pulmonary effects for the dose of 615 OTU (Sterk and Schrier, 1985). Other empirical calculation methods have been put forward. So for an average duration stated by the patients of 38±16 min at 1010±523 mbars of O2, the percentage chance of developing neurological signs linked to oxygen toxicity would be between 5% and 100%, with the average values giving 15% according to the OAA (National Oceanis and Atmospheric Administration) table, the limit of 100% being considered to be toxic for an individual producing little effort

(Hamilton Jr, 1997). Even though they are conservative, the latter a *minima* calculations do not address the medical treatments which use oxygen. We cannot effectively make an accurate estimation of the total quantity of oxygen inhaled without the dive profiles, and it is even more difficult to anticipate their effect. As the toxicity range seems wide, it would appear difficult to deny the possibility of a toxicity risk for the nervous system.

References

Brubakk, A.O., Neuman, T.S., and Elliott, D.H. (2003). *Bennett and Elliott's Physiology and Medicine of Diving, Oxygen Under Pressure by Clark, J.M. and Thom S.R.*: Saunders.

Hamilton Jr, R. (1997). Tolerating oxygen exposure.

Sterk, W., and Schrier, L. (Year). "Effects of intermittent exposure to hyperoxia in operational diving", in: *Research Inst. of National Defence The 11 th Annual Meeting of the European Undersea Biomedical Society(EUBS) on Diving and Hyperbaric Medicine p 123-131(SEE N 86-19854 10-52)*).

Wingelaar, T.T., Van Ooij, P.a.M., and Van Hulst, R.A. (2017). Oxygen Toxicity and Special Operations Forces Diving: Hidden and Dangerous. *Front Psychol* 8**,** 1263.
